# Investigating visual perception abilities in flight cadets: the crucial role of the lingual gyrus and precuneus

**DOI:** 10.3389/fnins.2025.1519870

**Published:** 2025-02-26

**Authors:** Xi Chen, Shicong Zhang, Shipeng Dong, Qingbin Meng, Peiran Xu, Qi Chu, Donglin Huang, Cheng Luo

**Affiliations:** ^1^Institute of Flight Technology, Civil Aviation Flight University of China, Guanghan, Sichuan, China; ^2^Aviation Health Department, Southwest Regional Administration of Civil Aviation Administration of China, Chengdu, China; ^3^Hospital of Civil Aviation Flight University of China, Civil Aviation Flight University of China, Guanghan, Sichuan, China; ^4^Key Laboratory for NeuroInformation of Ministry of Education, School of Life Science and Technology, University of Electronic Science and Technology of China, Chengdu, China

**Keywords:** fMRI, flying cadets, precuneus, visual perception, degree centrality

## Abstract

**Introduction:**

In aviation, exceptional visual perception is crucial for pilots to monitor flight instruments and respond swiftly to deviations, as well as make rapid judgments regarding environmental changes, ensuring aviation safety. However, existing research on pilots’ visual perception has predominantly focused on behavioral observations, with limited exploration of the neurophysiological mechanisms involved.

**Methods:**

This study aimed to investigate the brain activity associated with the visual perception capabilities of flight cadets. Data were collected from 25 flying cadets and 24 ground students under two conditions: a resting-state functional magnetic resonance imaging session conducted in 2022 and a change-detection task. The data were analyzed using RESTplus software.

**Results:**

The analysis revealed that degree centrality values in the right precuneus and left lingual gyrus showed significantly positive correlations with task reaction time and accuracy, respectively, in the pilot group. These brain regions were found to be significantly associated with the visual perception abilities of the pilots.

**Discussion:**

The findings suggest that alterations in the left precuneus and right lingual gyrus in pilots are linked to their visual perception capabilities, which may play a crucial role in mission performance. These results provide a foundation for improving flight training programs and selecting suitable flight trainees based on neurophysiological markers of visual perception.

## Introduction

In 2006, an aviation tragedy occurred when a Boeing 737 in Brazil collided with the Legacy 600 business jet, Despite changes to the status of the transponder display and the traffic collision avoidance system (TCAS) in the cockpit of the Legacy aircraft, the crew failed to detect these alterations. This incident serves as a critical reminder: during flight, particularly under “poor viewing conditions,” pilots often exhibit greater confidence in their visual capabilities than is warranted by their actual perceptual abilities. This discrepancy merits further discussion. Pilots engage in highly integrated flight tasks that necessitate awareness of numerous identical or similar stimuli that repeatedly manifest within their visual field over specific time intervals, requiring constant behavioral adjustments. The process of detecting changes in stimulus information represents an essential cognitive function in dynamic situations, especially during aircraft maneuvers (Wickens et al., 2009), and is closely related to a pilot’s visual perception ability (Lewkowicz et al., 2018). Visual perception ability encompasses a pilot’s skill to swiftly and accurately identify visual information amidst the complex environment of a cockpit; this capability is vital for monitoring instruments such as airspeed indicators, heading indicators, and altimeters while facilitating appropriate operational responses. When confronted with unexpected events, individuals process information through three primary mechanisms: situational attention, diagnosis, and response. Research conducted by Jones and Endsley (1996) on failures in pilots’ aviation situational awareness (SA) revealed that most failures transpired at the initial stage—attention and awareness—rather than at subsequent stages (Wickens et al., 2009; Ogawa and Macaluso, 2015). Consequently, precision in visual perception is paramount for maintaining situational awareness as well as for decision-making processes during flight; it directly influences aviation safety.

The flicker paradigm has been employed to investigate the capacity for change detection in experimental studies (Rensink et al., 2015). This experiment was structured by alternating between two slightly different images, with a temporal gap separating them, wherein the subtle changes serve as the target for detection. The findings indicate that when alterations in objects or scenes coincide with brief visual disruptions, observers typically fail to notice these changes, despite their existence. This phenomenon is referred to as “change blindness” (Simons and Levin, 2003). Previous research has established a connection between visual search and the activation of the frontal–parietal attention network; furthermore, activity within the prefrontal cortex is strongly correlated with successful identification of facial identity changes (Achaibou et al., 2016). During active searching and object detection within three-dimensional space, engagement of the prefrontal-parietal attention control network underscores the critical role that attentional mechanisms play in visual change detection (Ogawa and Macaluso, 2015). Moreover, disparity signals can enhance processing of complex scenes by modulating activity in the occipital visual cortex. Studies have demonstrated that neural responses in the lateral occipital cortex (LO) amplify traces of visual stimuli relevant to change detection (Schwarzkopf et al., 2010). Concurrently, neuronal activity within the medial temporal lobe (MTL) region has been closely linked to both change detection and change blindness (Reddy et al., 2006).

In recent years, rapid advancements in functional magnetic resonance imaging (fMRI) technology have enabled researchers to investigate the neural mechanisms underlying phenomena that occur during real-life activities. Previous fMRI studies have demonstrated that substantial cognitive training can enhance cognitive performance and modify factors related to brain function as well as morphological characteristics of the brain (Meyer et al., 2017; Baykara, 2021; Lerch et al., 2011). One study (Adamson et al., 2014) suggested that activation in the bilateral caudate nucleus is lower among expert pilots compared to those with moderate expertise, and that higher landing accuracy of expert pilots was associated with lower caudate activity. Another investigation utilized fMRI to reveal activation of the right dorsolateral prefrontal cortex (DLPFC) in pilots subjected to transcranial direct-current stimulation (Mark et al., 2023). These findings illustrate that fMRI technology has emerged as an essential tool for exploring brain activity, providing profound insights into the neural mechanisms underlying various cognitive processes, perceptual responses, and behaviors.

Degree centrality (DC) serves an fMRI index for evaluate voxel centrality by assessing the number of connections between a specific voxel and other voxels at the whole-brain level. A voxel exhibiting elevated DC values signifies its critical role in facilitating information transfer throughout the brain. This value is frequently employed to detect alterations in the brain network (Wen et al., 2022; Chen et al., 2023). The analysis minimizes the influence of subjective seed-site selection and, as a quantitative method to reveal the importance of nodes in a network, DC has shown potential in linking neurobiological substrates and professional skill performance (Zuo et al., 2012; Wen et al., 2022). Our previous study also indicated that flight training enhances DC values in both the left middle frontal gyrus and left lingual gyrus (Chen et al., 2023). In contrast to earlier resting-state fMRI studies that focused on measures of seed-based functional connectivity and evaluations of large-scale brain networks, an analysis employing voxel-wise degree centrality emphasizes the significance and impact that specific networks hold at the voxel level, delineating their central roles within the functional architecture of the brain (Li et al., 2016).

The primary hypothesis of this study posits that changes in DC within certain posterior neocortical regions among pilots may correlate with visual perception. We aimed to collect resting-state fMRI data from a cohort of experienced pilots, which, when combined with behavioral test scores from change-detection tasks, would enable us to investigate how attributes of brain networks relate to their professional efficacy. Our analysis is anticipated to provide substantial insights into pilot training and evaluation processes within the aviation industry.

## Materials and methods

### Participant recruitment

The experiment was approved by the Ethics Committee of the University of Electronic Science and Technology of China (Chengdu, China; No. 1420200408-07). All experimental procedures were completed in the Magnetic Resonance Imaging Center of the University of Electronic Science and Technology of China. All participants were informed of and understood the experimental process and signed informed consent before the start of the experiment. The experiment began on March 15, 2021 and ended on February 15, 2024.

We recruited flying cadets from the Civil Aviation Flight University of China. The criteria for selecting pilot cadets are as follows: (1) all candidates must be undergraduate students enrolled concurrently; (2) male; (3) completion of 40 h of simulation training is required; (4) practical flight training experience is necessary. The exclusion criteria for cadets include: (1) history of traumatic brain injury; (2) presence of neurological disorders; and (3) contraindications to MRI, such as claustrophobia or the presence of metal implants in the body. A total of 25 male cadets and 24 male ground students were included. The mean age of the cadets was 21.84 years (SD = 0.69), and the mean age of the ground students was 21.88 years (SD = 0.85). The cadets’ aircraft types are SR-20, DA-42 and C172R.

### Task procedures

**Figure 1:**
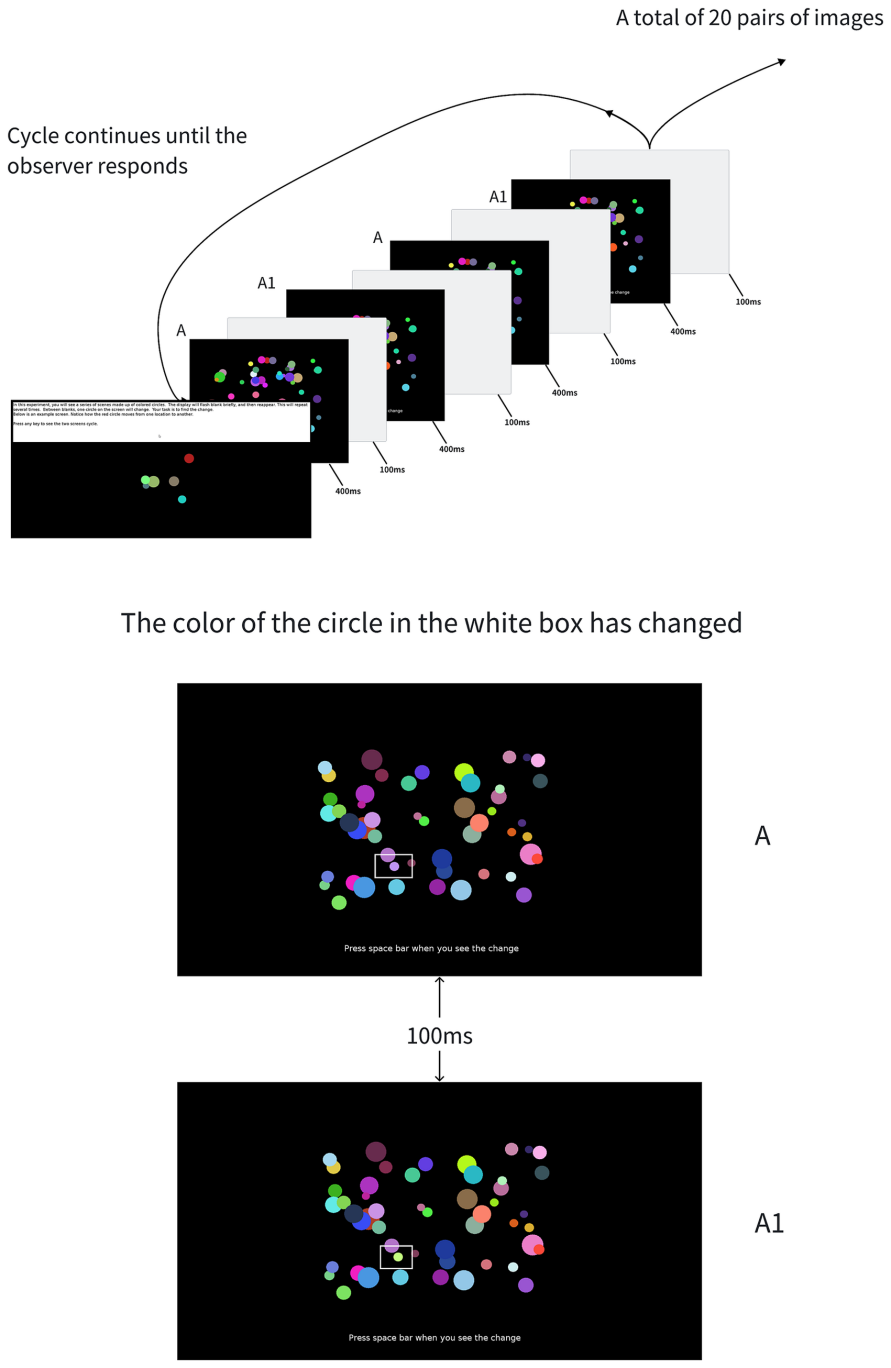
Overall design of change detection task. The experiment begins with a simple interface showing instructions, telling the user how to operate, and then enters the formal measurement after a simple operation by the user. A flashing sequence appears first and continues until the observer responds. In this example, the original image A (which has not modified the size of A circle) and the modified image A1 (which has modified the color of A circle) are followed by A, A1, A, A1, … The order of the display. There are gray areas between successive images.

The experiment was divided into two evaluation phases. First, the participants underwent a resting-state scan, during which they were required to close their eyes and relax. The next phase was conducted in the laboratory, where the participants were asked to complete a change-detection task on a computer using the PEBL2 software.^1^ The task began with the presentation of 20 pairs of images in succession (40 images in total, with an image size of 600 by 400 pixels), each displayed for 400 ms. Each image contained 50 circles of varying color (except black and white) and size (minimum radius is 10 pixels, maximum radius is 30 pixels). The pairs of images were presented in alternate order, with two images in each pair alternating at intervals of 100 ms, and the participant’s task was to identify changes in the presence, position, size, and color of the circles (each change was completely random and only changed one place. For example, when only the size of a circle is changed, its existence, position, and color will not change with other circles). The participants were instructed to press space key as soon as they noticed any change to record reaction time (RT) and then use the mouse to indicate the exact location of the change, at which point accuracy (ACC) was recorded and the system told the participant whether it was correct or incorrect (Figure 1). Before the experiment began, participants were first presented with instructions: “In this experiment, you will see a series of scenes made up of colored circles, and the screen will briefly flash blank and then reappear. In between the blanks, a circle on the screen will change. Your task is to find the change.” Participants began the experiment immediately after a training session. After participants made a choice between one pair of images, they moved on to the next pair of images until all 20 pairs were completed.

### Image acquisition

A GE 3.0 T MR750 magnetic resonance imager equipped with an 8-channel phased-array head coil was used. A standard gradient-echo pulse sequence was used. For T1 image acquisition, the specific scanning parameters were set as follows: field of view (FOV), 256 mm × 256 mm × 154 mm; matrix, 56 × 256; repetition time (TR), 5.976 ms; echo time (TE), 1.976 ms; flip angle, 9°; slice number, 154; the voxel size, 1 mm × 1 mm × 1 mm. For fMR image acquisition, the specific scanning parameters were set as follows: flip angle, 90°; slice thickness, 4 mm (no interval); image matrix, 64 × 64; FOV, 240 mm × 240 mm × 140 mm; (TR)/echo time (TE), 2000 /30 ms. Each participant was scanned in 8.5 min, each scan comprised 35 slices of the whole brain, and a total of 255 frames were collected from the whole brain.

Before entering the scanning room, the participants underwent a whole-body check for metals and were assisted in wearing earplugs. To prevent excessive head moves due to involuntary subtle movements, foam cushions were utilized to stabilize the head, and the lights were immediately turned off. Participants were positioned in the scanning device and asked to lie down with their eyes shut. They stayed alert, do not fall sleep and were given directions to refrain from any particular or consistent mental tasks.

### MRI data preprocessing

The resting state fMRI data were preprocessed using the SPM12^2^ and RESTplus (Jia et al., 2019)^3^ software based on the MATLAB R2013b platform. Data preprocessing was based on the following steps: ① conversion of the DICOM data format to NII; ② removal of the first five time points. ③ slices timing and head-motion correction for the remaining 250 scans; ④ spatial normalization—individual brain images were registered to the Montreal Neurological Institute (MNI) template, with a standardized space of 3 mm × 3 mm × 3 mm; and ⑤ removal of linear drift and bandpass filtering (0.01–0.08 Hz). The signals of head - motion parameters (rigid body 6 parameters), white matter, and cerebrospinal fluid were regressed as nuisance covariates.

### Degree centrality analysis

After preprocessing the resting fMRI time series data, we utilized the RESTplus software to conduct voxel-by-voxel DC analysis. The method allows calculation of the average Pearson correlation coefficient between a single voxel and all other voxels in the brain, thereby this approach enables assessment of the significance of each voxel as a node in the network. This value is commonly used to detect changes in the brain network. Finally, we construct a Pearson correlation coefficient between any two voxels in the brain, notably taking only positive correlations above the *r* = 0.25 threshold.

Generally, there are two methods to calculate DC: weighted DC (wDC) and binarized DC (bDC). Because weighted DC considers the weights of edges, the analysis is usually continued with the values of weighted DC.

Based on the adjacency matrix of the graph, voxel DC can be calculated as follows (Zuo et al., 2012; Liao et al., 2013).

$$DC\left(1\right)=\overset{N}{\underset{j=1}{\sum }}a_{ij}$$

Where a_ij_ is the correlation coefficient between voxel I and voxel j.

After DC was calculated using RESTplus, the Fisher z-transformed results were further processed and the z-score maps were smoothed using a 6-mm full-width half-maximum Gaussian kernel.

## Statistical analysis

The data were analyzed on the MATLAB R2013b platform, and the RESTplus toolkit was used for the statistical calculation of the DC values for each participant. The DC values of all voxels in the two groups were calculated using the independent sample t test Pearson correlation analysis was performed between the DC values of all voxels per participant and ACC and RT of the corresponding individual. The results were corrected using Gaussian random field (GRF), with a clustering level of *p* < 0.05 and initial height threshold of *p* < 0.001.

## Results

### Participants and behavioral results

The results showed that 25 flying cadets and 24 ground students were eventually obtained, and no one was excluded because of excessive head movement. Table 1 shows the demographic parameters and behavioral results, showing the mean and standard deviation of age, gender, education, total flight time, change detection_total accuracy, and change detection_ reaction time, respectively.

**Table 1:** Demographic parameters and the results of change detection task.

	Flying cadets (*N* = 25)		Ground students (*N* = 23)		Significance	
	M	SD	M	SD	*t*-value	*p*-value
Age (years)	21.84	0.69	21.88	0.85	−0.159^a^	0.88
Gender (%male)	100%		100%			
Education (years)	12		12			
Total flight time (simulation flying) (hours)	40	0				
Change detection_total accuracy (%)	92.40	4.72	91.90	9.529	0.61	0.54
Change detection_reaction time(s)	22310.60	8035.41	20257.07	6675.54	0.97	0.34

### Correlation analysis

**Figure 2:**
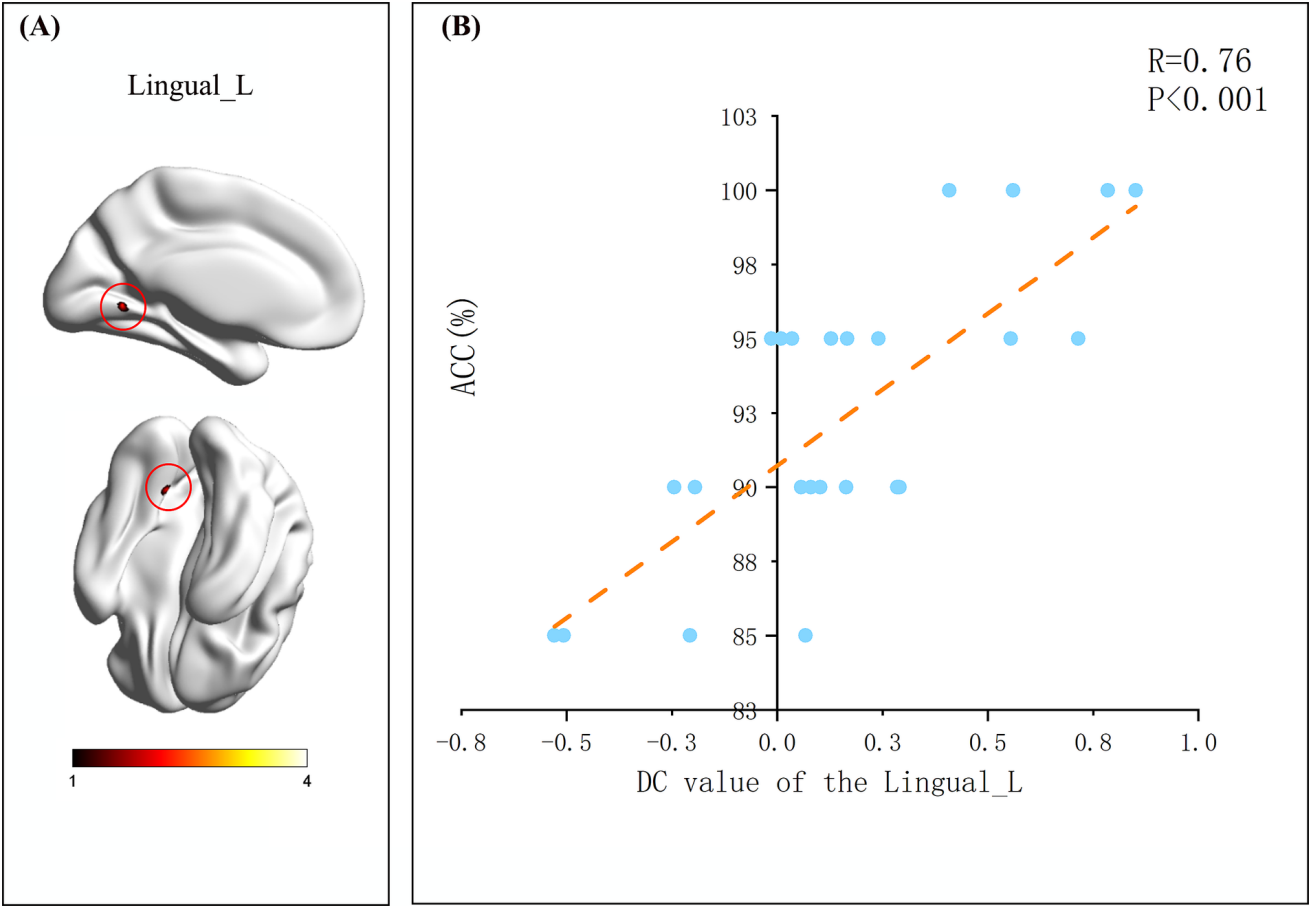
Details on accuracy. **(A)** Represented brain regions with significant associations (grf correction, *p* < 0.05, initial height threshold, *p* < 0.001). The smaller the value of the color bar label, the more significant the difference between the two groups of participants in the brain region of this location. **(B)** The results indicated that there was a significant correlation between the DC value of Lingual_L and ACC (*p* < 0.05). Each circle represents a subject’s degree centrality value and task performance, specifically the relationship between degree centrality value and accuracy (ACC) of the left lingual gyrus. The orange dotted line indicates the corresponding linear regression trend.

**Figure 3:**
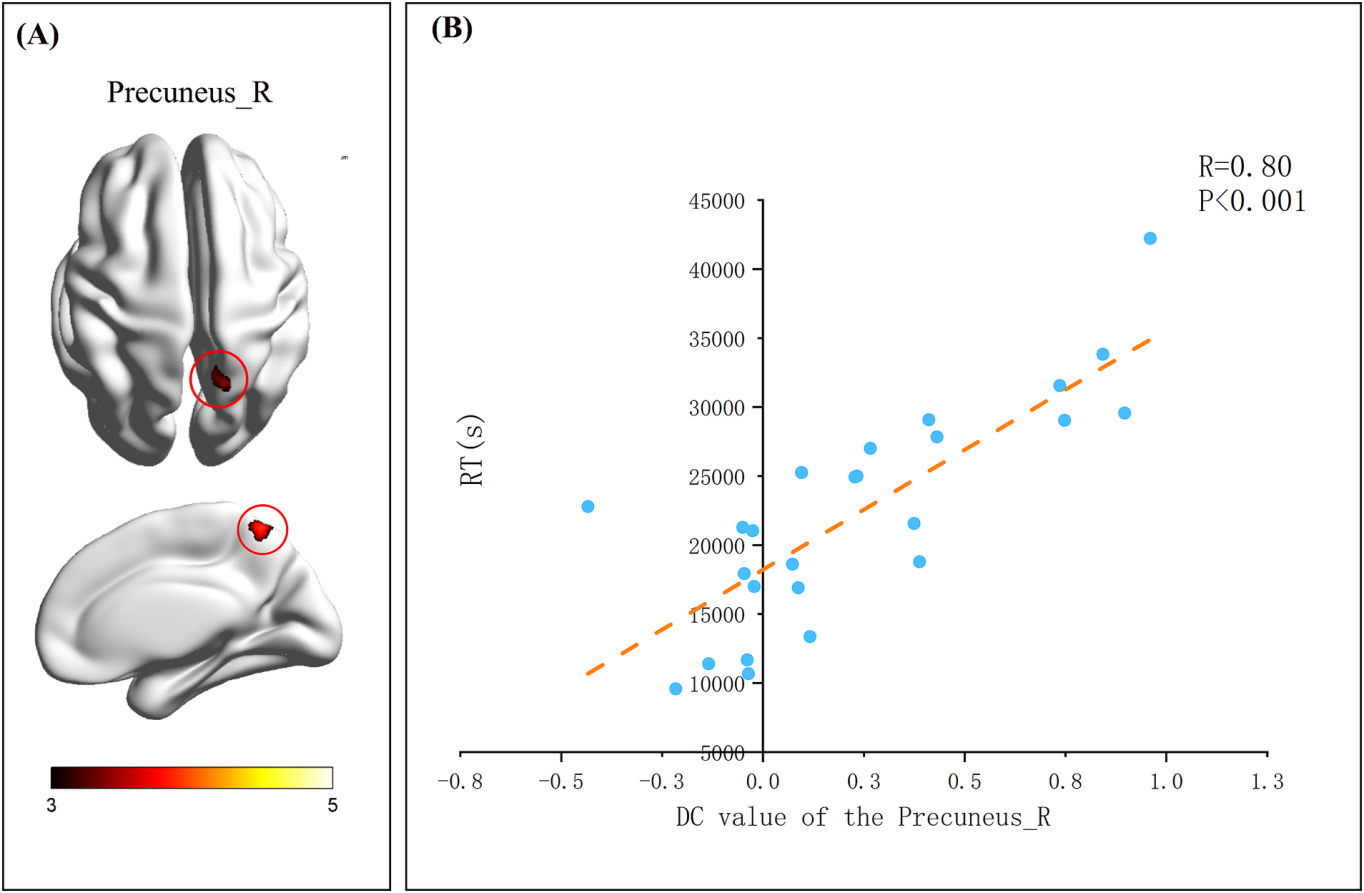
Details on react time. **(A)** Represented brain regions with significant associations (grf correction, *p* < 0.05, initial height threshold, *p* < 0.001). The smaller the value of the color bar label, the more significant the difference between the two groups of participants in the brain region of this location. **(B)** The results indicated that there was a significant correlation between the DC value of Precuneus _R and RT (*p* < 0.05). Each circle represents a subject’s degree centrality value and task performance, specifically the relationship between degree centrality value and reaction time (RT) of the right precuneus. The orange dotted line indicates the corresponding linear regression trend.

**Figure 4:**
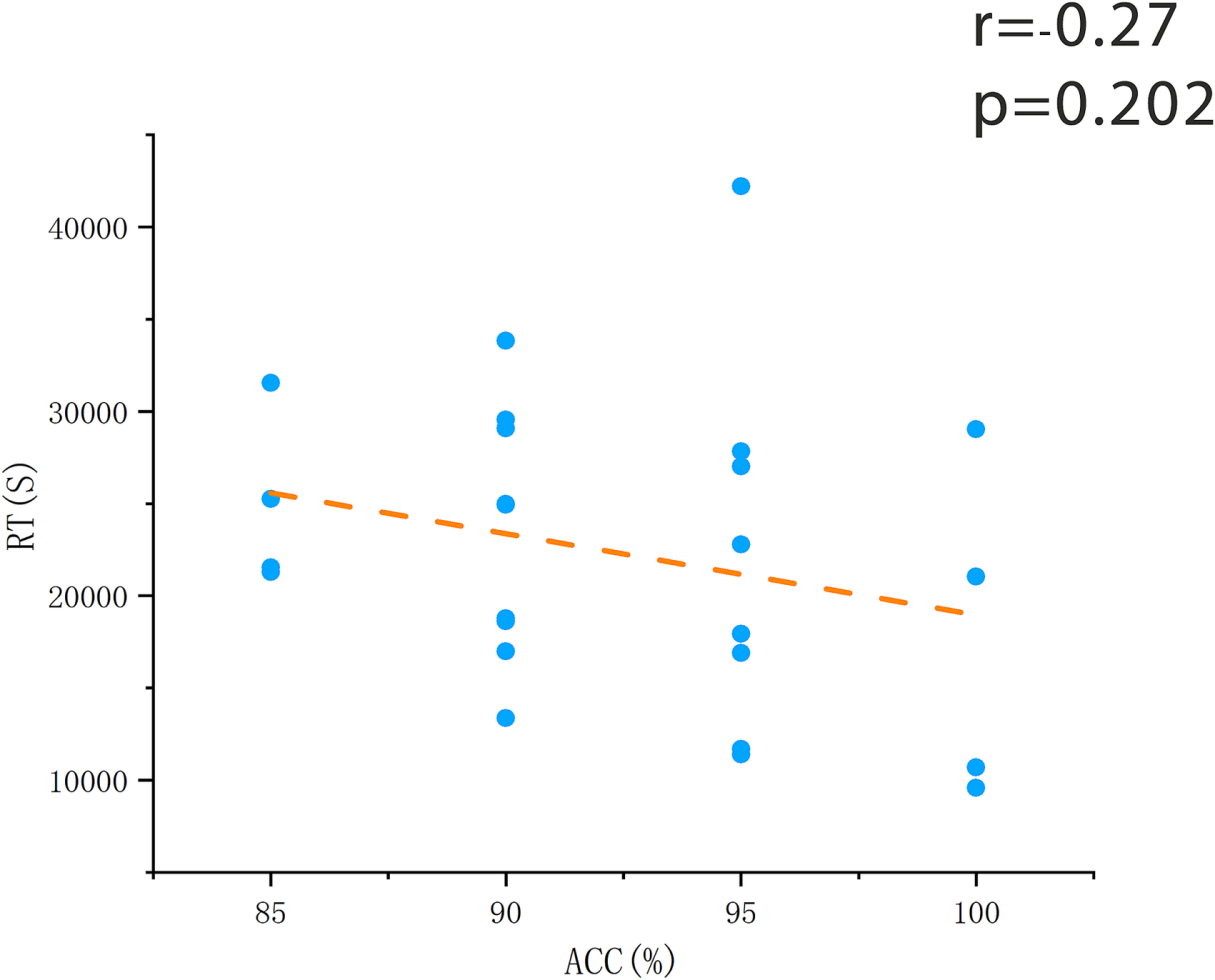
The correlation between reaction time and time. The results showed no significant correlation between RT and ACC in the cadet group (*p* = 0.202). Each circle represents a participant’s RT vs. ACC, and the orange dotted line represents the corresponding linear regression trend.

**Figure 5:**
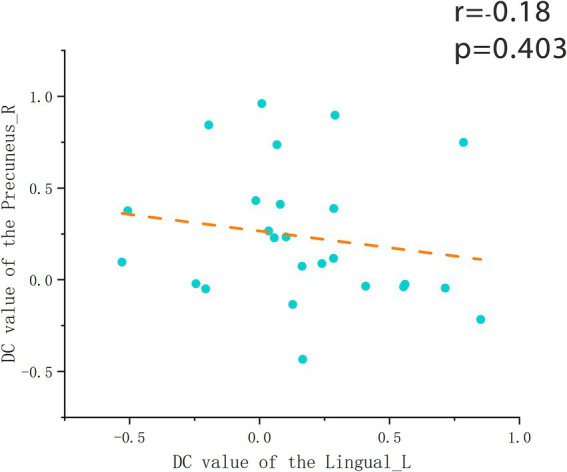
Correlation of DC values of right precuneus and left lingual gyrus. The results showed that there was no significant correlation between the DC values of the right precuneus and the left lingual gyrus in the cadet group (*p* = 0.202). Each circle represents the DC values of a participant’s right precuneus and left lingual gyrus, with orange dashed lines indicating the corresponding linear regression trend.

After assessing whole-brain degree centrality (DC), we performed an independent sample t-test of DC values for each brain region between the two groups, and Pearson correlation analysis between the DC values of each region and the indicators of change detection task. The results showed no significant differences in brain regions or behavioral data between the two groups. In the flying cadets group (Table 2), a significant positive correlation was found between left lingual gyrus DC values and ACC (*r* = 0.76, *p* < 0.001; Figure 2). In addition, the DC values in the right precuneus were significantly positively correlated with RT (*r* = 0.799, *p* < 0.001; Figure 3). Furthermore, within the flying cadets group, there appears to be a correlation between ACC and RT (*r* = −0.27, *p* = 0.202; Figure 4), though not statistically significant. Similarly, there also appears to be a trend with negative correlation between the DC values of the left lingual gyrus and right precuneus (*r* = −0.18, *p* = 0.403; Figure 5), but the results were not statistically significant.

**Table 2:** Brain region information significantly related to the change detection task indicators of flight trainees.

Brain region	Voxel number	MNI coordinates			*t*-peak value
		x	y	z	
Precuneus_R	111	21	−60	48	4.6246
Lingual_L	116	−9	−57	−3	4.1296

## Discussion

In this study, we used fMRI to study the brain regions closely related to the visual perception of pilots. By implementing the visual change-detection task, we revealed a significant correlation between changes in the response of specific brain regions and the visual perception ability of the pilots. Specifically, the results showed a high positive correlation between the lingual gyrus and ACC, as well as between the precuneus and RT. This finding indicates that when pilots perform change-detection tasks based on visual perception, the state of activity in the right precuneus and left lingual gyrus areas will affect the speed and accuracy of visual information processing and may be related to overall flight performance.

Flying tasks involve complex cognitive skills, excellent visual perception ability is of vital significance for ensuring flight safety and for improving mission execution. Previous research has indicated that the lingual gyrus plays a crucial role in visual processing when the brain receives stimulation signals. Specifically, the lingual gyrus is involved in both basic and advanced visual processing tasks related to stimuli direction and color (Palejwala et al., 2021). Functional studies have also demonstrated that activation of the lingual gyrus is preferentially observed when words are presented with low contrast, and is involved in direction and motion discrimination (Cornette et al., 1998; Zhang et al., 2016). Consequently, pilots who exhibited higher levels of reaction in the lingual gyrus during this process likely display increased sensitivity for changes in visual stimuli, leading to higher ACC scores. At the same time, lingual gyrus is closely related to visual memory. Patients with bilateral ischemic stroke affecting the lingual gyrus exhibit impaired visual memory and an association between the storage of visual memory and the volume of gray matter in the lingual gyrus, suggesting a potential functional link between visual memory and this brain region (Bogousslavsky et al., 1987; Zhang et al., 2019). In summary, our findings align with prior research indicating that neural coding processes related to visual imagery may be enhanced within the region encompassing the lingual gyrus when stimuli are during the execution of a change detection task. These investigations have underscored the role of posterior brain regions in motor imagery (Malouin et al., 2003), as well as contributions to visual memory (Zhang et al., 2016) and representation (Olivetti Belardinelli et al., 2009).

The results of this study found that higher activity levels in the precuneus were associated with longer reaction times, while activity in the left lingual gyrus tends to decrease. This suggests that pilots may develop highly automated operational patterns through training and experience, making their actions and decisions more efficient and streamlined (Steinman, 2023). The precuneus also plays an important role in visual attention conversion, visual information integration, and spatial memory. Studies have shown that the precuneus is associated with shifts in attention to novel visual stimuli (Le et al., 1998), and this structure can also process the transfer of attention between object features (Nagahama et al., 1999). The findings of this study are consistent with these observations in that the perception of changes in the characteristics of parts of a visual stimulus is associated with the precuneus. In addition, activity in the precuneus engages an extensive network of cortical and subcortical structures that coordinate and integrate information flow from higher centers (Tanglay et al., 2022; Messina et al., 2023), rather than simply representing responses to external stimuli. At the same time, the precuneus connects the geodesic fibers to the adjacent parietal and occipital cortices, including the parietal occipital visual area and the PO2 region of the parietal cingulate network, which has been shown in previous studies to have extensive connections with the occipital visual cortex, and the lingual gyrus is a part of the visual cortex, mainly involved in visual information processing and visual memory. Especially the secondary visual cortex (V2, V3) (Bogousslavsky et al., 1987; Cavanna and Trimble, 2006; Tanglay et al., 2022; Dadario and Sughrue, 2023). A previous study investigated which brain structures have a distinct role in the retrieval of contextual memory using a word pairing association test, and found that bilateral activation in the precuneus was associated with contextual memory retrieval through direct comparisons with semantic retrieval (Shallice et al., 1994). In addition, a classic study (Fletcher et al., 1995) examined possible links between the precuneus and visual performance by analyzing memory strategies employed during memory retrieval. The results showed that memory-related visual imagery was associated with significant activation of the bilateral precuneus, confirming that this is a key structure for visual imagery during episodic memory recall (Stephan et al., 1995). All the above studies indicate that the precuneus plays an important role in attention conversion, visuospatial memory and episodic memory extraction, and visual information integration, and that activity in this medial structure provides the neural basis for the pilots’ visual perception ability.

This study has some limitations. First, we did not use images of flight scenes as experimental material, which compromised ecological validity. Second, we only collected fMRI data in the resting state and did not investigate the relevant parameters in the task state. Third, the high accuracy of task performance may present some challenges to the interpretation of results. Lastly, larger sample sizes and longer resting state scanning times, such as 30–60 min, are needed to confirm the results of this study. Subsequent research can be improved by expanding the scope and increasing the number of research subjects. Ecological validity is increased by using images of flight scenes as experimental materials, such as the use of aircraft alarm signals. Future research could consider adopting more challenging or different difficulty change detection tasks, or further exploring other types of tasks. Incorporate individuals of different age groups and various professional backgrounds yet with similar cognitive - ability characteristics. Compare the activities and potential changes in brain regions during the same or similar tasks among them. Alternatively, collect data on change detection under task - state MRI. Through these means, more comprehensive and universally applicable supporting evidence for research viewpoints can be provided.

In conclusion, our study found that, in pilots who perform the change-detection task, the DC values in the lingual gyrus and the precuneus significantly positively correlated with ACC and the RT response, respectively. We speculate that the response of these brain regions is related to the visual perception ability of the pilots. We hope that the results of this research will be the basis for constructing more scientific flight training plans and selecting flying cadets. In the process of pilot psychological selection, brain imaging technology can be used to evaluate the activation of relevant brain regions when candidates perform visual search tasks. In terms of training, the research findings can provide a basis for the formulation of personalized training programs. For example, the activation of the precuneus was enhanced through simulated tasks, thereby improving the pilot’s reaction speed and attention regulation.

## Data Availability

The datasets presented in this study can be found in online repositories. The names of the repository/repositories and accession number(s) can be found at: https://pan.baidu.com/s/1O-yS5Zw-2YjHpa3nlHXbLQ?pwd=xvaa.

## Footnotes

^1^ https://pebl.sourceforge.net/

^2^ https://www.fil.ion.ucl.ac.uk/spm/

^3^ http://www.restfmri.net/forum/restplus
